# A Case of Acute Inflammation of the Sublingual Glands

**Published:** 1849-01

**Authors:** Edward M. Boykin

**Affiliations:** Camden, S. C.


From Charleston Medical Journal and Review.

### A CASE OF ACUTE INFLAMMATION OF THE SUBLINGUAL GLANDS.

BY EDWARD M. BOYKIN, M. D.—*Camden, S. C.*

WEDNESDAY, March 29th.—W. E., a North Carolina wagoner (a white man,) was brought to me for examination; he seemed suffering great pain—his throat much swollen externally; not being able to articulate at all, or swallow any thing. The cavity of the mouth was completely filled up, with what appeared to be the tongue in a swollen state; but on close examination, the principal swelling was found to be in the sublingual glands, which were enormously distended, and, rising up from their bed, were pressing against, and on a level with the lower teeth, forcing the tongue against the roof of the mouth. The tip of the tongue was curled up, and turned as far back as its attachments would permit. Its superior surface could not be seen. There was no getting any thing into the stomach.

He had been perfectly well in the morning, and had eaten his breakfast on the road, as usual; after eating, he felt uncomfortable about the mouth and throat, which feeling rapidly increased, so that by the time I saw him, 4 P. M., some eight or nine hours after feeling the first unpleasant symptoms, he was in the state described above. It would appear, that the day before, the patient was actively engaged in loading his wagon in Columbia, being anxious to get out of town that day, and making a day's drive; in doing which, he exerted himself very much, and drove on until 9 o'clock at night, walking before his horses with a lighted torch in his hand. After getting to his camping ground, he took several large draughts of cold spring water in the heated and excited state in which he then was; he slept all night, as is the custom of his class, under no covering but his blanket, in the open air. The year before, he had suffered from a violent attack of pneumonia, making it necessary, as he said, "to put a blister on him as big as the skirt of his saddle."

The inflammatory symptoms running high, and the feeling of suffocation, momentarily increasing, I bled him ad deliquium. I then cut deeply into the glands on both sides; they bled freely, and for a short time, immediately after, he seemed slightly relieved, but was wholly unable to swallow, and soon became as bad off as before. The incisions were repeated twice, each time with temporary relief, although not much blood followed: leeches could not be obtained. Some clots of ropy mucus were with difficulty drawn from behind the tongue, which appeared to relieve the overwhelming sense of suffocation in some degree. He then fell asleep, sleeping and dozing for an hour or more; between 8 and 9 o'clock he was able to swallow a little water from a spoon, by drawing down the end of the tongue and putting the spoon over it; the tongue had become more flexible, but still, when left to itself, the tip turned upward; 12 grains calomel with 20 grains jalap were administered; a compound liniment of ammonia and iodine, was rubbed on the external swelling, and a poultice put over it. The parotid gland seemed, as well as could be determined, free from inflammation.

March 30.—Found him in the morning more comfortable; the medicine had acted freely in the night; he could speak a little, and swallow without a spoon, but painfully, and with great difficulty; the incisions under the tongue were discharging freely a semi-transparent yellowish fluid, the consistence of the white of an egg; this was encouraged by washing out the mouth with warm milk and water; the external swelling had increased, extending down the entire length of the neck; slight cough. Kept up the action of the bowels with Epsom salts; continued poultice and liniment.

March 31st.—Comparatively comfortable since last seen, had taken a little gruel, been freely purged; the tip of the tongue had resumed its normal position, and its upper surface could be seen; it had a dark red streak on each side, and a coat of yellow fur down the center. Complained of pain in his right side, and cough more decided; the swelling of the throat had abated a good deal.

April 1st.—Swelling of the throat gone down almost entirely, the tongue its natural size, incisions in glands healed over. But pain in the right side very severe, and cough oppressed and troublesome, a feverish flush in the face, pulse not much accelerated, but showing some excitement; covered seat of pain with a blister; gave anodyne expect. mixt.

April 2nd—Blister drawn well, the pain in a great measure relieved, cough less, the lungs relieved by free expectoration; bowels kept open, patient much weakened.

April 3rd.—Continued to improve—got gradually better, and though still very weak, left for home, near Charlotte, North Carolina, on the 5th of April.

From its similarity to the case cited below, the term *Ranula* might have been applied here, as in that case, although the symptoms were strictly inflammatory, the swelling receding with the inflammation; the patient not complaining of any previous enlargement under the tongue, or noticing the gradual increase of the gland; which would have been the result of an obstructed duct. The case appeared peculiar, and is submitted as possessing some points of interest.

CASE.—"The elder Cline used to mention in his lectures, that he was one morning alarmed, by the noise of a person breathing with great difficulty, in the next room to his consulting room; and on hastening in, he found the man stretched on a chair, and almost suffocated. On being inquired of as to what was the matter, he pointed to his mouth—upon looking into which, Cline observed a large *Ranula* thrusting back the tongue, which he instantly punctured with a lancet, and relieved the patient from threatened suffocation."—*Chelius, by South*, p. 121, vol. iii.

